# Supplementary material for: DEAD box RNA helicase 5 is a new pro-viral host factor for Sindbis virus infection
Source: Virol J. 2024 Mar 29;21:76. doi: 10.1186/s12985-024-02349-3 (PMC10981342; doi:10.1186/s12985-024-02349-3)
Supplement: Supplementary file 2 — Supplementary Material 2. [file 12985_2024_2349_MOESM2_ESM.pdf]

A

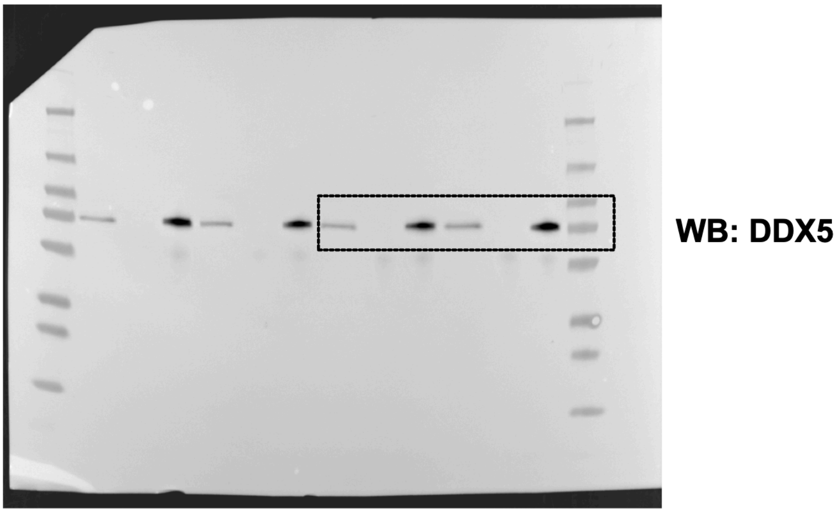

D

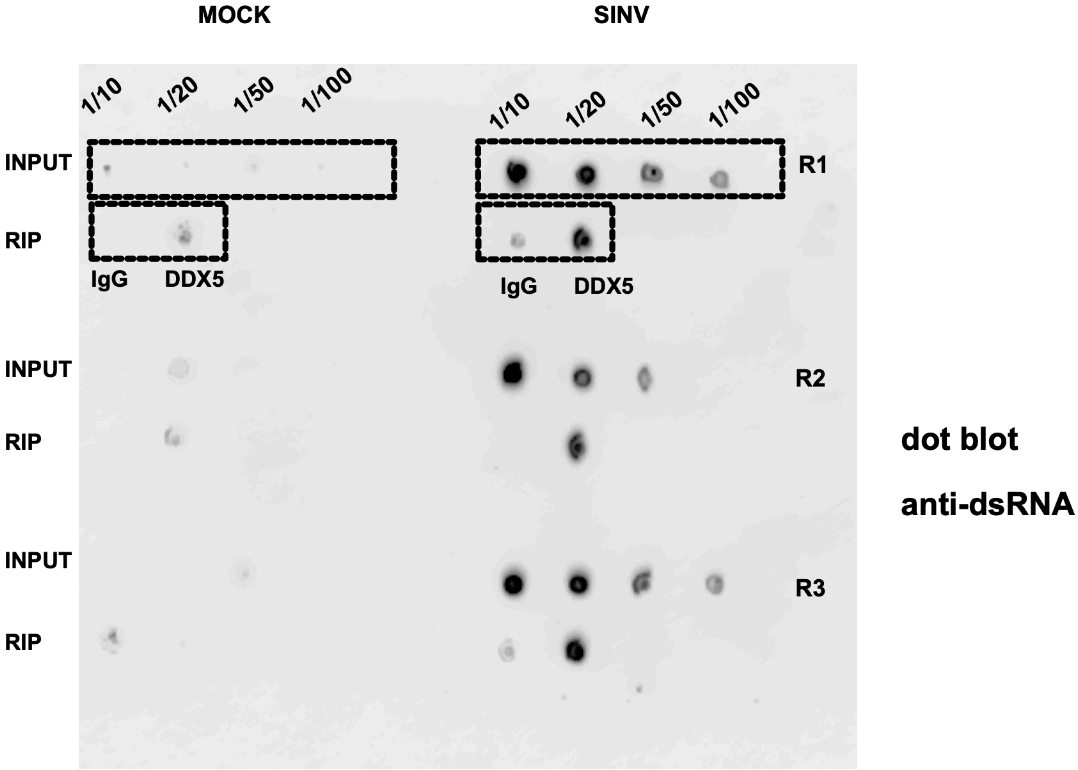

Figure S1 (related to Figure 1)

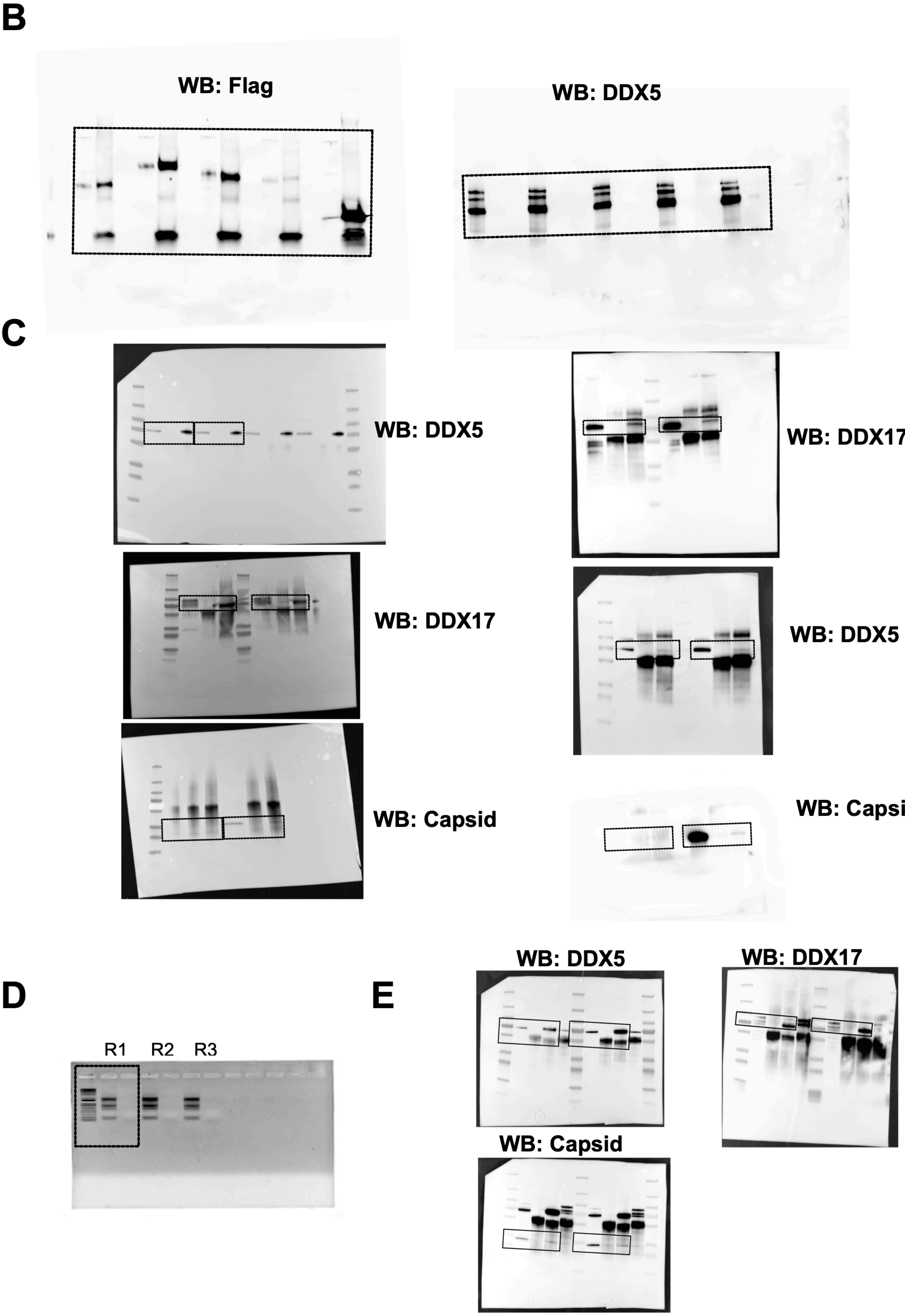

Figure S2 (related to Figure 3)

A

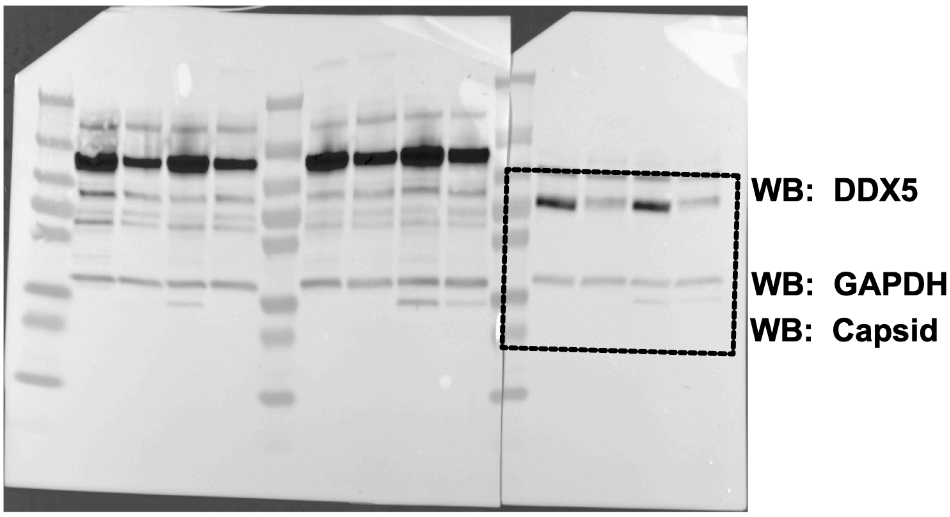

E

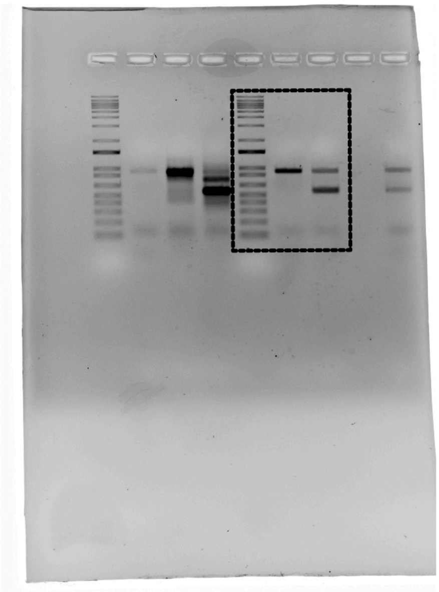

F

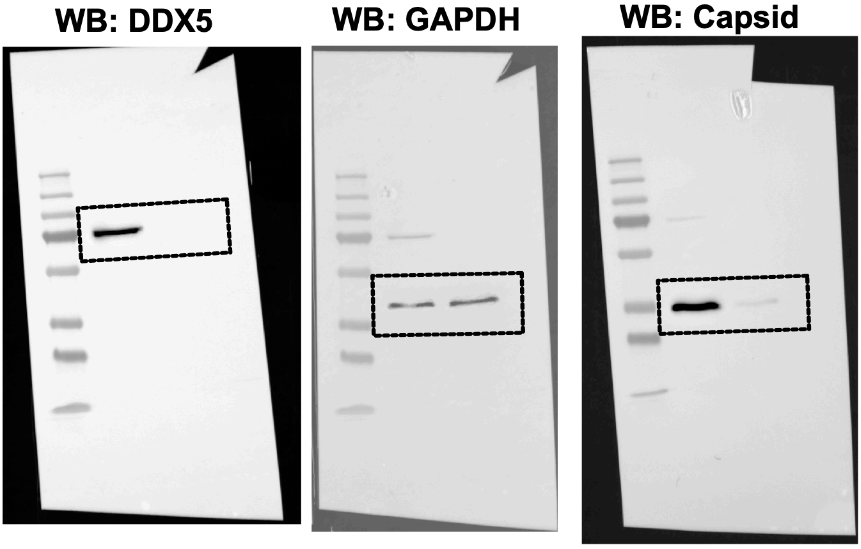

Figure S3 (related to Figure 4)

A

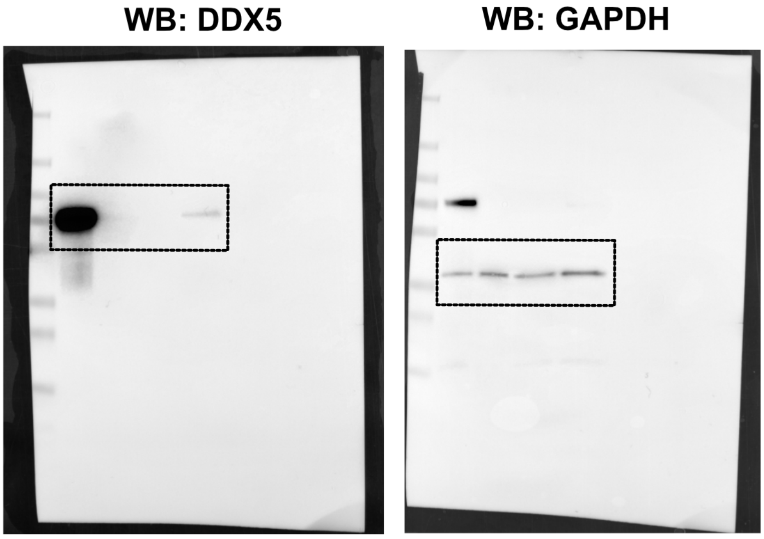

B

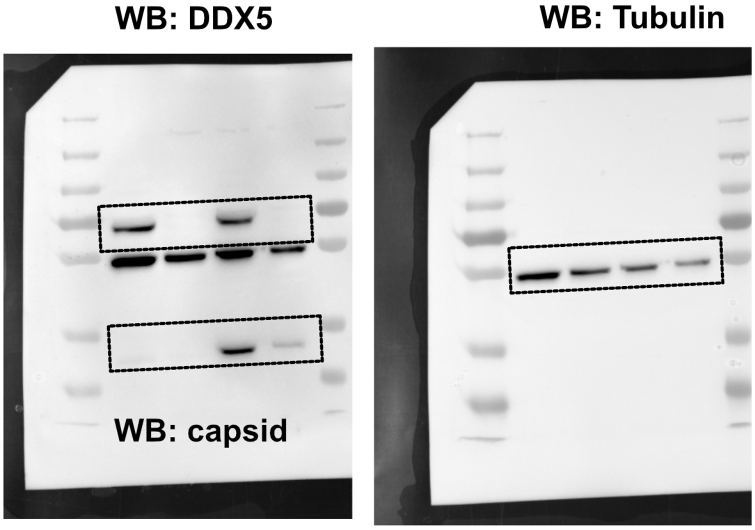

C

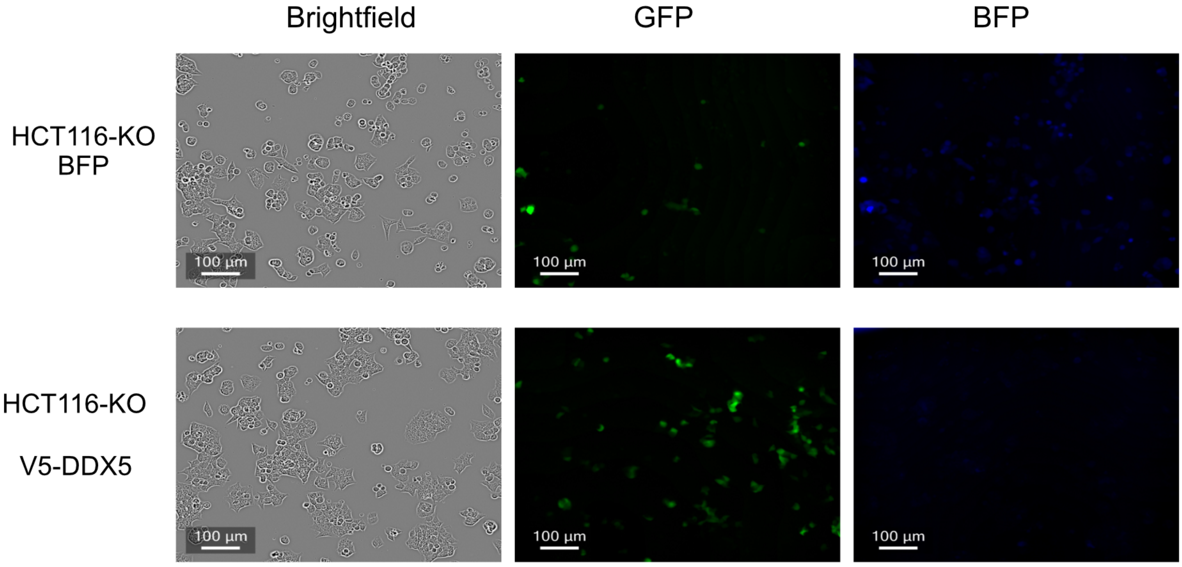

Figure S4 (related to Figure 5)

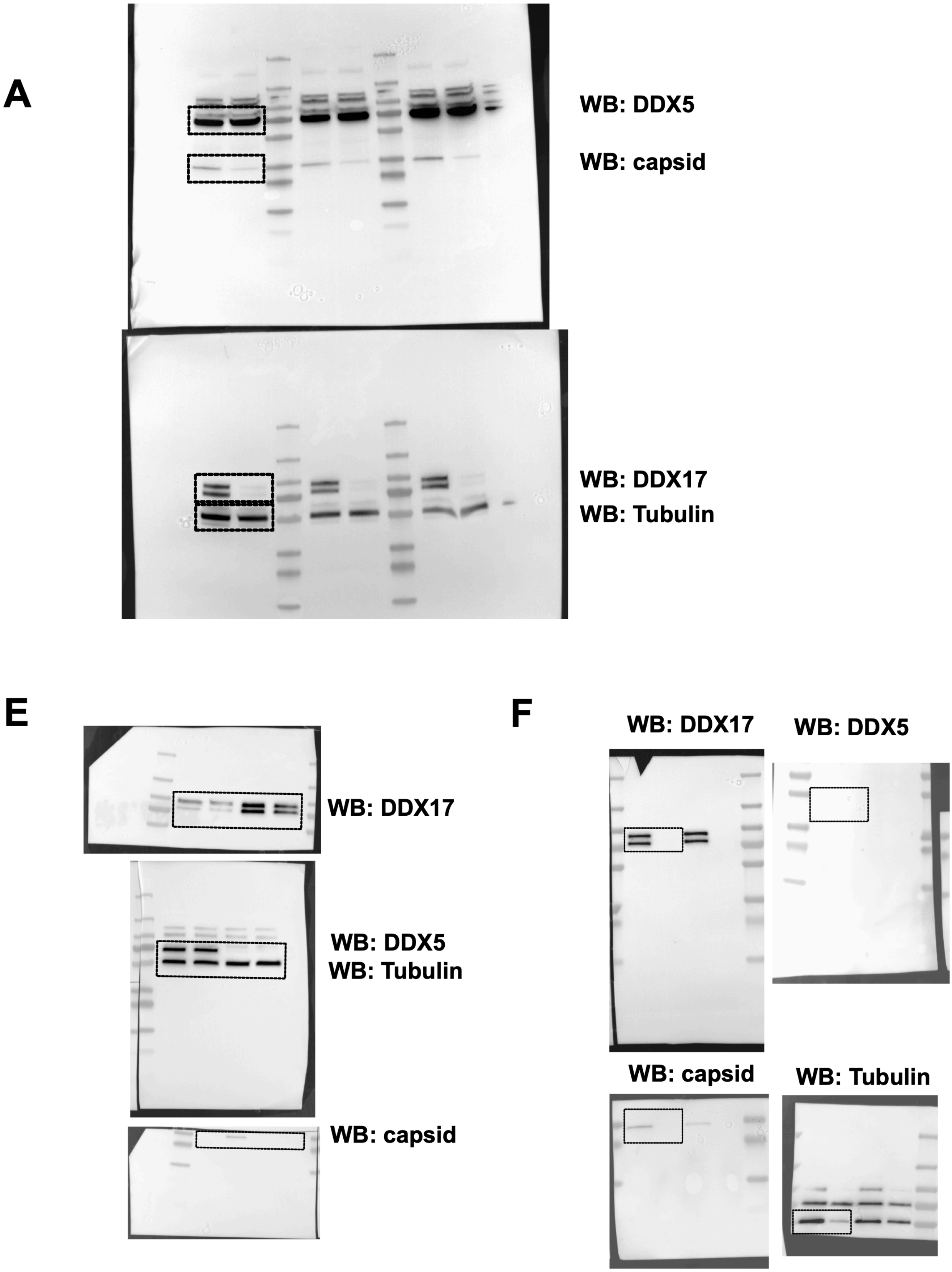

Figure S5 (related to Figure 6)
